# Polarization-Sensitive Super-Resolution Phononic Reconstruction of Nanostructures

**DOI:** 10.1021/acsphotonics.1c01607

**Published:** 2022-05-18

**Authors:** Rafael Fuentes-Domínguez, Shakila Naznin, Salvatore La Cavera III, Richard Cousins, Fernando Pérez-Cota, Richard J. Smith, Matt Clark

**Affiliations:** † Optics and Photonics Group, University of Nottingham, University Park, Nottingham NG7 2RD, United Kingdom; ‡ Nanoscale and Microscale Research Centre, University of Nottingham, University Park, Nottingham NG7 2RD, United Kingdom

**Keywords:** super-resolution, phonons, nanostructures, pump−probe, time-resolved

## Abstract

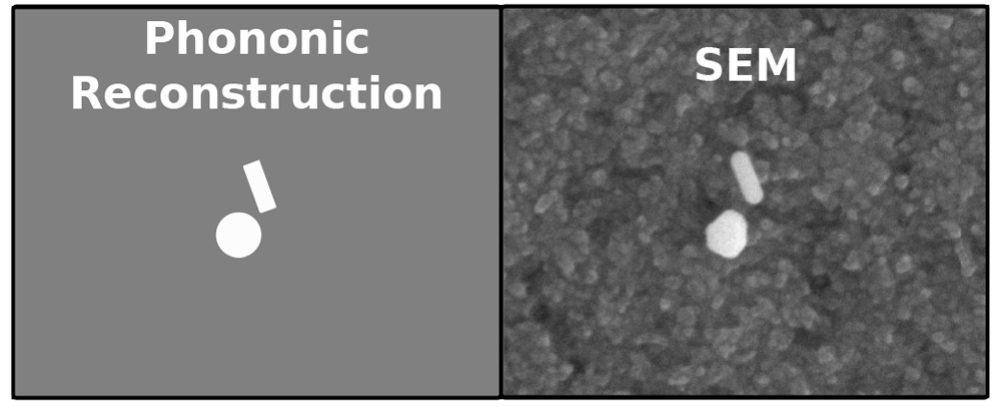

In this paper, we show for the first time the polarization-sensitive super-resolution phononic reconstruction of multiple nanostructures in a liquid environment by overcoming the diffraction limit of the optical system (1 μm). By using time-resolved pump–probe spectroscopy, we measure the acoustic signature of nanospheres and nanorods at different polarizations. This enables the size, position, and orientation characterization of multiple nanoparticles in a single point spread function with the precision of 5 nm, 3 nm, and 1.4°, respectively. Unlike electron microscopy where a high vacuum environment is needed for imaging, this technique performs measurements in liquids at ambient pressure, ideal to study the insights of living specimens. This is a potential path toward super-resolution phononic imaging where the acoustic signatures of multiple nanostructures could act as an alternative to fluorescent labels. In this context, phonons also offer the opportunity to extract information about the mechanical properties of the surrounding medium as well as access to subsurface features.

## Introduction

Super-resolution fluorescence microscopy has had a tremendous impact in life sciences over the last two decades for its ability to reveal the insights of biological processes at nanoscale dimensions.^1−4^ This technology mainly relies on the switchable states of fluorophores (on and off) after being activated by light. For instance, stimulated emission depletion (STED)^5,6^ microscopy works by optically “turning off” the fluorophores around the imaging point using a ring-shape pattern of light, which leaves an active area that is smaller than the optical diffraction limit. Furthermore, photoactivated localization (PALM)^7,8^ and stochastic optical reconstruction (STORM)^9,10^ microscopy achieve super-resolution by stochastically switching and repeatedly localizing the photoactivate states.

However, fluorophore-based detection schemes suffer from two major drawbacks: they require typically high light intensities, which can produce optically induced damage,^11^ and the fluorophores bleach, losing the ability to emit light over time.^12^ Therefore, alternatives to fluorescence labels are aiming to overcome these limitations. For instance, it has been shown that a single nano-object detection is possible through nonfluorescent approaches such as spatial modulation spectroscopy (SMS)^13,14^ and photothermal microscopy.^15,16^ SMS works by modulating the analyte’s position relative to a diffraction-limited probe and allowing the acquisition of a single particle’s extinction spectrum across the visible and near-infrared. Alternatively, photothermal microscopy, also known as thermal lens microscopy, detects the small additional divergence of a transmitted probe beam through the heating-induced thermal lens, i.e., the refractive index gradient around a heated nanoparticle.

All the above methods rely on the optical properties of either fluorophores or nanostructures to provide super-resolution. However, using phonons also permits one to achieve similar goals through time-resolved pump–probe spectroscopy. This technology has enabled the study of ultrafast events in metallic nanostructures such as electron dynamics,^17,18^ electron–phonon coupling^19,20^ and phonon dynamics.^21−25^ The first two effects occur in the very fast time regime, i.e., hundreds of femtoseconds, whereas the phonon dynamics occur from hundreds to several thousands of picoseconds. This technology and the phonon dynamics provide a method to characterize simultaneously the size and shape of simple structures such as spheres and rods,^26^ as well as the provide super-resolution imaging capabilities on spherical nanoparticles^27^ with a precision of 3 nm. Moreover, phonon technologies offer a way to extract information on the mechanical properties of the surrounding medium.^28^ This has led to its application to cell biology in an effort to increase our understanding of normal and disrupted cell physiology.^29,30^

In this paper, we demonstrate for the first time that polarization-sensitive time-resolved pump–probe spectroscopy can reconstruct multiple nanostructures with different shapes under the same optical point spread function (PSF). The ability to control the polarization of light allows the orientation characterization of nonspherical nanostructures, where the detection of a nanorod can be turned on and off by varying the probe laser polarization. Hence, it is possible to obtain full characterization of these structures, including size, shape, angle orientation, and localization, and all in a biocompatible environment (water). With this technique, we can achieve characterization similar to that possible using scanning electron microscopy (SEM),^31^ but without the need of high vacuum environments or additional metallic coating, and establish a path toward imaging living cells with electron microscopy resolution.

## Experimental Setup

**Figure 1:**
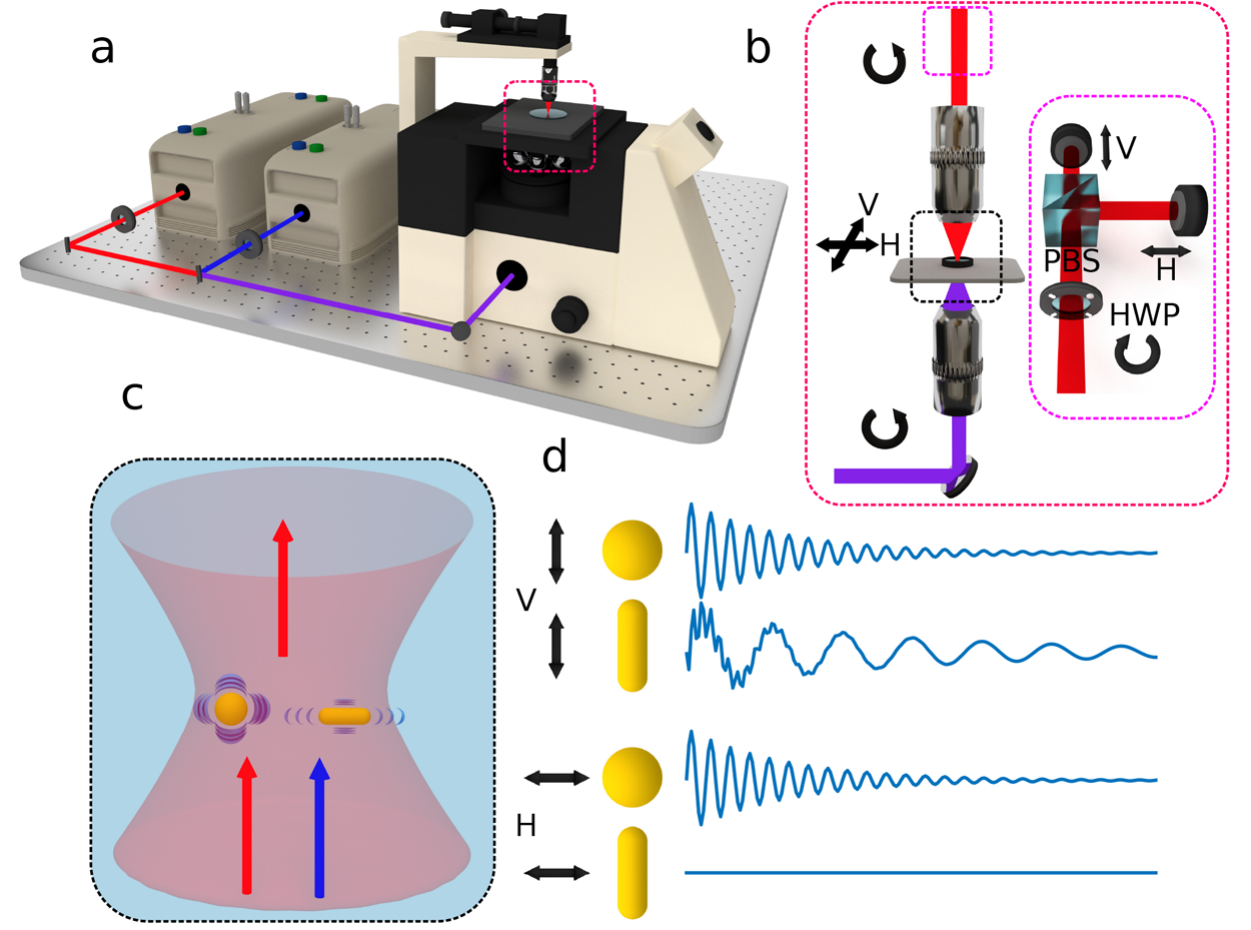
(a) Simple schematic of the experimental setup. (b) Magnified area of the sample stage with input and output circular polarized light, which is modified by a half-wave plate (HWP) and polarizing beam splitter (PBS) (inset). This allows simultaneous measurement of the horizontal (H) and vertical (V) axis at the sample plane, as well as their rotation with the HWP (ϕ_Sample_ = 2ϕ_HWP_). (c) Diagram of the optical point spread function (PSF) with a sphere and a rod vibrating. (d) Simulated mechanical response of a sphere (single frequency) and a rod (extensional and breathing mode frequencies) with linear polarized light. Here, it can be seen that the detected rod vibrations can be turned on and off, which allows the orientation characterization.

The time-resolved pump–probe spectroscopy setup is built around a dual Ti:sapphire (Tsunami Spectra-Physics) laser asynchronous optical sampling system (ASOPS) with 100 fs pulses at an 80 MHz repetition rate.^27^ The pump and probe lasers (415 and 780 nm, respectively) are delivered to an inverted microscope and focused to the sample plane by an objective lens (0.45 numerical aperture, NA), which produces an optical point spread function of ∼1 μm (fwhm) and limits the optical resolution to around 1 μm. Additional polarization elements are mounted above the optical deck (Figure 1a) consisting of an automatic rotational half-wave plate, a polarizing beam splitter, and two photodetectors. This permits the detection of both cross-polarizations at the sample stage plane simultaneously. We have chosen that the polarization axis of the half-wave plate matches the horizontal axis or 0 degrees at the sample stage.

Because the time-resolved measurements are performed in water, the substrate was treated with an electrostatic layer-by-layer self-assembly process to adhere the nanostructures such that they do not wash away. A detailed description of this process is shown in the Supporting Information. Then, the sample was placed in a gasket cell filled with deionized water and mounted on a positioning stage with 100 nm step size for scanning (Figure 1b).

The polarization detection setup (Figure 1b) consists of a half-wave plate (HWP), a 50:50 polarizing beam splitter (PBS), and two detectors. The system is calibrated in a way that when the HWP is at 0 degrees the detected probe light matches the horizontal (H) and vertical (V) axes at the sample plane. By rotating the HWP angle (ϕ_HWP_), the H and V axes rotate following this expression: ϕ_Sample_ = 2ϕ_HWP_.

Figure 1c shows a diagram of spherical and rod nanostructures inside an optical PSF. The pump laser (415 nm) excites the nanostructures, which causes the mechanical vibration, and this is measured with very high precision by the probe laser modulation (780 nm). Both pump and probe light arriving at the sample are circularly polarized, but only the transmitted probe light, after interacting with the nanostructures, is propagated through the polarization detection setup (HWP and PBS). Additionally, the measured nanostructures are insensitive to the pump laser polarization, and the maximum variation in the extinction cross-section due to light polarization (or orientation) is in the near-infrared wavelengths (see Supporting Info).

Examples of the signal detected from the sphere and rod are shown in Figure 1d. When the half-wave plate axis, or probe laser polarization, is along the rod length (vertically), the detected rod response is turned on. Conversely, the rod response is off when the polarization aligns with the rod width. It is important to note that the sphere is insensitive to any polarization because of its spherical symmetry.

### Detection of Nonspherical Nanostructures

**Figure 2:**
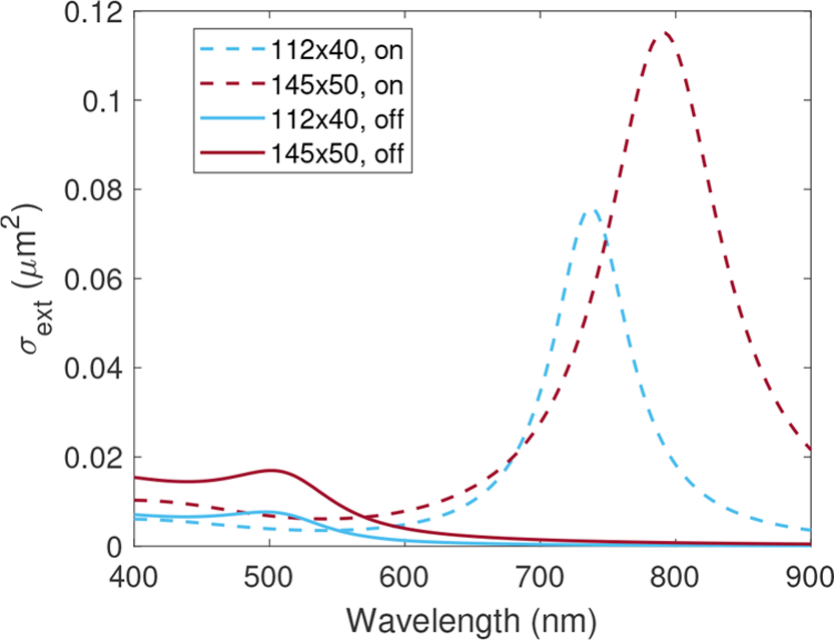
The extinction cross-section for two rods (112 × 40 and 145 × 50 nm) when the light polarization is along the length (on) or along the width (off). The probe laser wavelength is 780 nm.

To understand how the change in probe light polarization can provide orientation information on nonspherical nanostructures, we have simulated the extinction cross-section^32^ of the gold nanorods used in this work: 112 × 40 and 145 × 50 nm, length and width, respectively, in water. The optical spectrum shown in Figure 2 varies depending on the light polarization. Here, the maximum amplitude is obtained when the light polarization matches the rod length (on) and the minimum is measured axially, along the rod width (off).

Therefore, we can obtain 360 deg angle information by sweeping the probe light at the near-infrared wavelengths (780 nm here) for different polarization angles. In our experimental setup, this is done by rotating a half-wave plate after the sample, which is more stable than rotating polarizing optics before the sample, affecting the laser overlapping and reducing the SNR.

This detection mechanism not only allows us to measure the orientation of the nanorods but also opens a way to reconstruct, with super-resolution, nanorods with the same vibrational frequencies (same size) when their orientations are different.

It should be noted that in our experimental measurements all nanostructures are constantly excited by the pump laser (415 nm), i.e., the phononic modes are being continuously generated. For the particular case of nonspherical nanostructures, the nanorod phononic modes are not detected when the optical extinction cross-section is minimal (Figure 2), turning off the detection as shown in Figure 1d. This is only possible (with the sizes presented here) by using near-infrared wavelengths for detection.

### Size, Shape, Position, and Orientation Measurements

**Figure 3:**
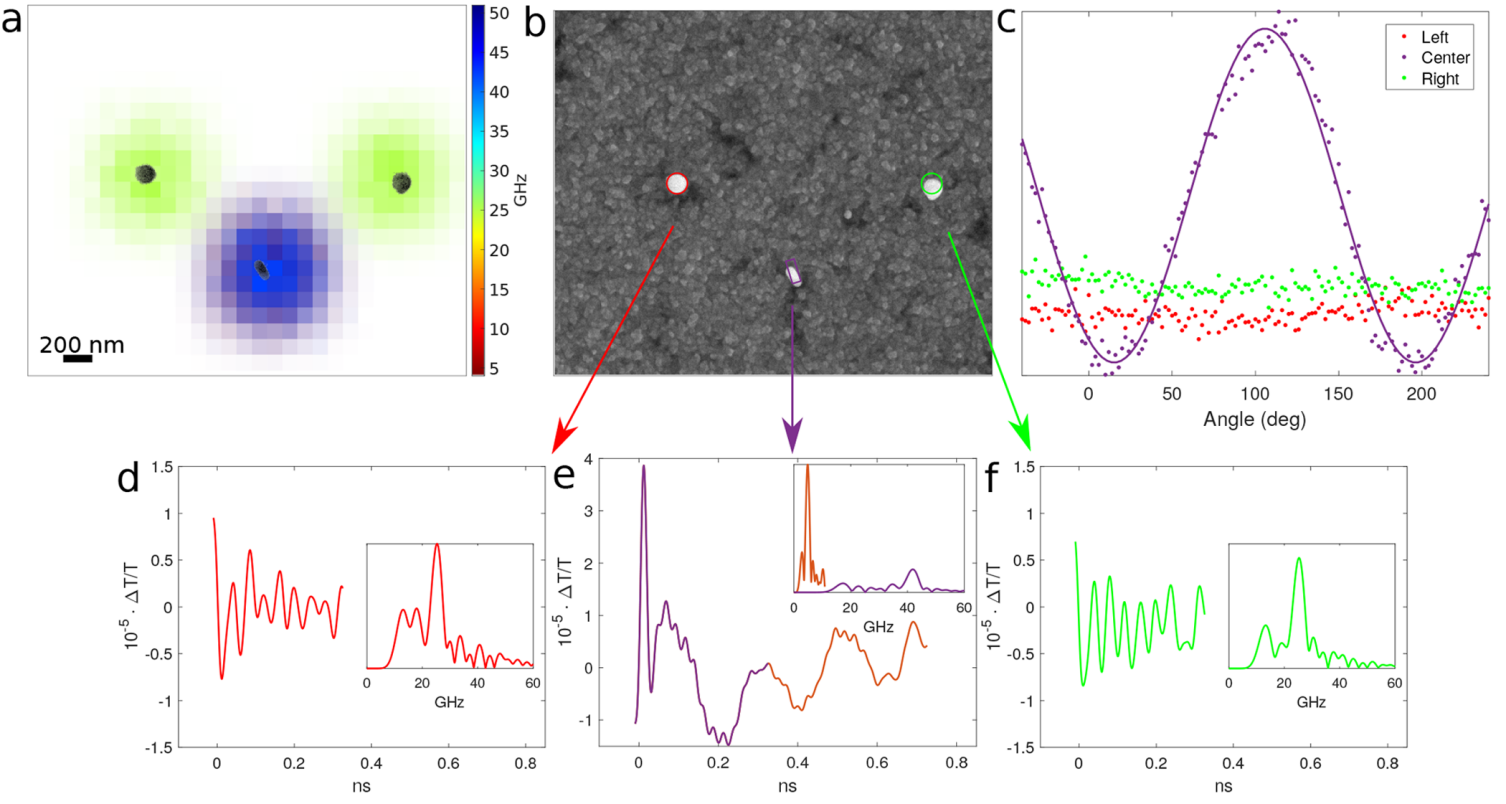
The phononic reconstruction method. (a) Overlay between SEM and the frequency map where a single frequency is measured on a sphere (green), whereas two modes are obtained on a rod (red + blue = purple). (b) Phononic reconstruction of the size, shape, orientation, and localization with the SEM overlaid. (c) Angle characterization plots where the sphere is insensitive to linear light polarization and the rod matches with the real orientation maximum signal at 107°. (d–f) Time and frequency traces from each optical point spread function.

The reconstruction of the size, shape, position, and orientation is extracted by the time-resolved measurements. First, the vibrational frequency of each nanostructure is obtained by applying a fast Fourier transform (FFT) to the time domain signal after removing the electronic excitation occurring at *t* = 0 and the thermal background by fitting and subtracting a polynomial function. In the frequency domain, a single frequency is obtained for a nanosphere and two for a nanorod. As there is a large range of frequencies, time windows of varying length were used to isolate the frequencies of interest. Therefore, by plotting these frequencies at each pixel, one can obtain Figure 3a where two spheres and one rod are present in the scanned area, which shows a way to characterize the size and shape. Examples of the time and frequency traces for each nanostructure are shown in Figure 3d–f.

The position of each nanostructure is obtained by centroiding the FFT amplitude for each characteristic frequency and is detailed in Table 1. For the nanorod case, we have chosen the low frequency component to calculate the centroid because the SNR is slightly better due to lower attenuation. The acoustic data is compared with an SEM image in Figure 3b, and it can be seen clearly that all reconstructed parameters (size, shape, position, and orientation) are in very good agreement.

**Table 1:** Measured Frequency and Equivalent Size, Estimated Size Calculated from the SEM Images, and Angle Orientation from the Three Nanostructures in Figure 3^a^

nanostructure	frequency (GHz)		size (nm)		size SEM (nm)		angle (deg)
(1) sphere	23.54 ± 0.33		138 ± 6		125 ± 5		
(2) rod	4.87 ± 0.06	42.01 ± 0.48	150 ± 10	56 ± 2	140 ± 5	64 ± 5	107 ± 1
(3) sphere	25.27 ± 0.35		128 ± 5		125 ± 5		

a The size error values are the contribution of the dominant experimental error (i.e., the frequency error) and other factors such as the assumptions made about shape, elasticity constants, and environment effect. These values and assumptions are discussed in the Size, Shape, Position, and Orientation Measurements section.

The last parameter obtained in our setup is the nanostructure orientation. In this case, by rotating the detected probe light polarization (i.e., the half-wave plate at the detection arm), the maximum amplitude is obtained when the polarization angle matches that of the long axis of the nanorod. The amplitude variation for the example case is shown in Figure 3c. The signal amplitude of both spheres is constant with respect to polarization angle as spherical nanostructures are not sensitive to polarization. However, the nanorod amplitude variation shows a maximum and minimum when the light polarization is detected along the length and width, respectively.

The orientation can be also obtained by other detection mechanisms like rotating the input probe laser polarization before the sample. However, there is an additional challenge to this method, which is the alignment of both lasers at the sample stage. Rotating the waveplate to change the polarization state can move the probe beam at the sample plane, but because of imperfections in the waveplate and alignment of the optics, the signal amplitude could be reduced (if both lasers are not overlapping correctly at the sample stage), the SNR could be lowered, and even the wrong angle characterization could be obtained.

The size, position, and orientation precision of this technique were obtained as follows:

- The size precision was derived from the frequency precision (the frequency standard deviation of each measured nanostructure using the recorded data during the angle characterization) and converted using an analytical expression shown in the “Mechanical Vibrations of Nanostructures” section of the Supporting Information.

It may appear that the nanostructures are placed on a hard elastic layer; however, the surface treatment to fix the particles so that they remain in place when in water appears to acoustically isolate them from the substrate. In contrast, when nanostructures are built via electron-beam lithography (EBL) directly on the substrate, they show additional vibrational frequencies that are not present for the structures shown here.
Additionally, the effect of the surrounding environment (air or water) is only detected with the change in attenuation, but our method is insensitive to this as it does not change the frequency significantly (see Supporting Information). Therefore, we have assumed that the damping due to water is not significant in our measurements to characterize the size and shape.
From the above assumptions, the size precision values are shown in Tables 1 and 2, but generally our technique resulted in 5 nm size precision for the largest structures measured here.

- The localization precision was estimated by measuring the standard deviation of the signal level in regions with no structures and using this as a measure of the signal noise. This error was then used with simple error propagation analysis to estimate the position error of the calculated centroids, and these calculations were confirmed by simulation of signals with random noise added. This gave an estimation of ∼3 nm for the typical signals shown in the results.
- The orientation angle precision was estimated by measuring the standard deviation of the signal level in regions where the nanorod response is off and using this as a measure of the signal noise. Following the same process as the localization precision, this gave the angle precision to be 1.4°.

**Table 2:** Measured Frequency and Equivalent Size, Estimated Size Calculated from the SEM Images, and Angle Orientation from the Four Scans in Figure 4^a^

area	nanostructure	frequency (GHz)		size (nm)		SEM size (nm)		angle (deg)
(a)	rod	5.49 ± 0.07	40.06 ± 0.14	133 ± 9	59 ± 2	150 ± 9	56 ± 9	–6.1 ± 1.4
	sphere	24.39 ± 0.35		133 ± 4		122 ± 9		
(b)	sphere	24.88 ± 0.31		130 ± 3		124 ± 9		
	rod	5.23 ± 0.04	38.41 ± 0.18	140 ± 9	61 ± 2	159 ± 9	75 ± 9	22.2 ± 1.4
(c)	rod (left)	7.63 ± 0.04	67.18 ± 0.70	96 ± 6	35 ± 2	94 ± 4	34 ± 4	40.9 ± 1.4
	rod (right)	7.19 ± 0.07	63.06 ± 0.6	102 ± 7	37 ± 2	97 ± 4	37 ± 4	78.8 ± 1.4
(d)	sphere	22.22 ± 0.19		146 ± 3		138 ± 3		
	rod	4.74 ± 0.08	43.24 ± 0.88	154 ± 10	54 ± 2	162 ± 3	59 ± 3	98.6 ± 1.4

a The size error values are the contribution of the dominant experimental error (i.e., the frequency error) and other factors such as the assumptions made about shape, elasticity constants, and environment effect. These values and assumptions are discussed in the Size, Shape, Position, and Orientation Measurements section.

Combining the size, shape, position, and orientation information from the scanned area, we can obtain the phononic imaging reconstruction of multiple nanostructures with very high resolution and in very good agreement with SEM images.

### Super-Resolution Phononic Reconstruction

The previous steps were applied for a group of nanostructures further away than a single optical PSF. However, the same method can be replicated to obtain super-resolution phononic reconstruction of multiple structures with the same precisions.

**Figure 4:**
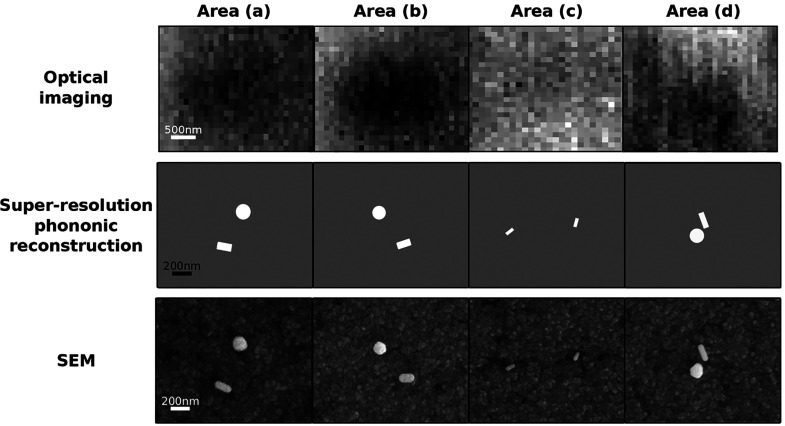
Optical, super-resolution phononic reconstruction and SEM images of multiple nanostructures inside the same optical point spread function.

Figure 4 shows a comparison between the optical picture, super-resolution phononic reconstruction, and SEM images of four different areas with multiple nanostructures inside a single PSF. While the optical imaging of the system has poor contrast (notice the larger scale bar, 500 nm), the super-resolution phononic images are in very good agreement with the SEM. The frequency, size, and angle orientation data are shown in Table 2 for the different areas. Here, we demonstrate that if the frequency of each nanostructure can be distinguished, we can obtain super-resolution phononic reconstruction images.

Being able to resolve multiple frequency peaks inside the same PSF is crucial to achieve super-resolution phononic images; therefore, nanostructures that share the same vibrational frequency (or channel) cannot be distinguished. However, adding polarization characterization offers a way to separate the same frequencies at different orientations and overcome the main limitation of this technique. This is shown in Figure 4c, where two rods with very similar dimensions (and frequency, less than 0.5 GHz) can be precisely characterized and superlocalized because their orientations are different.

The reconstruction obtained by our technique is in very good agreement with electron microscopy. Although the precision values were shown and discussed before, we have estimated the position accuracy by calculating the difference between the centroids of our reconstruction images and the SEM ones. This value is about 25 nm, which is equivalent to a quarter of a pixel in our time-resolved setup. However, this has to be treated carefully because the relative scaling of the SEM and optical images was performed using the scale bars in the SEM images, and the positional information from the microscope stage can add to this mismatch. Also, while performing the time-resolved measurements, the nanostructures are immersed in water, which could move slightly because of water pressure or temperature differences, and drifting of the whole sample could occur, induced by air bubbles forming in the sample chamber and increasing the difference with respect to the SEM images. We tried to reduce these risks by degassing the water to remove all air bubbles and shortening the acquisition time for each scanned area.

## Discussion and Conclusions

In this paper, time-resolved pump–probe spectroscopy offers an alternative to electron microscopy for characterizing and imaging groups of nanostructures beneath the optical diffraction limit of the setup (∼1 μm). This is accomplished by identifying each nanostructure with their vibrational frequencies, which allows us to get a full reconstruction: size, shape, position, and orientation.

Metallic nanostructures can be easily functionalized and well tolerated by biological cells.^33−35^ We propose then to use nanostructures as probes in a similar way as fluorophores in superoptical resolution techniques (like STORM or PALM). In this way, well-characterized structures (with known elastic properties as well as shape and size distribution) would be functionalized to tag specific proteins inside a cell to enable the mapping of, for instance, the cell membrane and nucleus with phononic resolution. This new scheme may offer significant advantages for cell applications over techniques based on fluorescent dyes. Nanostructures will not bleach, which will allow long-term, repeated imaging. Additionally, phonons will not cause damage, even though they have suboptical wavelengths, and will provide local mechanical information about the surrounding environment (i.e., viscosity or sound velocity). Although the nanostructures shown here are relatively large, their sizes can be reduced drastically to allow better cell intake without further drawbacks and keep similar optical properties in the near-infrared wavelengths (nanorods are ideal for this).

Although this technique offers potential advantages for living-cell imaging, there are still some challenges. The acquisition speed is not yet practical for biological applications, however, it could be increased, for instance, through wide-field detection. Also, the nanostructure sizes are relatively large compared with the PSF of the optical system but these were selected for ease of use, and much smaller particles can be measured.

In addition to cell imaging, we believe that this technique might open a way to deconstruct more complex structures made from simple shapes and fully characterize them by measuring their size, shape, localization, and orientation with very high precision. The super-resolution phononic reconstruction of nanospheres and nanorods demonstrated here would be equivalent to lines and dots in structures fabricated by electron-beam lithography. We believe that this is key to extend this technology beyond primitive shapes and enable the characterization of 3D nanometric structures, for example, in integrated circuits. Although this is beyond the scope of this paper, other geometrical shapes like triangles, squares, or pentagons (or their 3D geometrical counterparts: pyramids, cubes or dodecahedrons) could be reconstructed using the same experimental setup, but this also requires further modeling to understand the vibrational modes of these structures and complex fabrication requirements.

Furthermore, this technique would also enable measurements of the Young’s modulus and density at the nanometer scale, which can be related to the full elastic constant matrix. Although prior knowledge of the size and shape is needed, the phononic measurements could be combined with additional optical techniques, new models, and machine learning to acquire the full elastic information.

All this can only be achieved if we are able to separate each individual vibrational frequency from the nanostructures inside the PSF, which is not possible when imaging structures with very similar sizes. However, this has been overcome, as shown in Figure 4d, by varying the probe laser polarization. Here, we were able to separate the two longitudinal nanorod frequencies within the frequency resolution of our setup.

In conclusion, we have demonstrated for the first time the polarization-sensitive super-resolution phononic reconstruction of multiple nanostructures by using their characteristic vibrational frequencies. Here, time-resolved measurements allow one to characterize the size, shape, localization, and orientation of nanospheres and nanorods by overcoming the optical diffraction limit of the setup (∼1 μm). We have shown the capabilities of this technique as an alternative to electron microscopy without the need of high vacuum environments or additional coatings, while also offering a living cell potential by measuring these nanostructures in water. In addition to this, acoustic measurements can also provide information about the mechanical properties of the medium and might offer a way to obtain biomechanical information at the nanoscale.

## Supporting Information Available

Details of the fabrication process, the extraction of the nanostructure dimensions by analytical expressions, SEM imaging, image reconstruction and localization, noise and localization error estimation, polarization sensitivity to pump and probe, and further experimental traces (PDF)
