# Supplementary material for: Polarization-Sensitive Super-Resolution Phononic Reconstruction of Nanostructures
Source: ACS Photonics. 2022 May 18;9(6):1919–25. doi: 10.1021/acsphotonics.1c01607 (PMC9204812; doi:10.1021/acsphotonics.1c01607)
Supplement: Supplementary file 1 — ph1c01607_si_001.pdf [file ph1c01607_si_001.pdf]

Supporting Information: Polarization Sensitive Super-Resolution Phononic Reconstruction of Nanostructures

Rafael Fuentes-Domínguez,[*,†] Shakila Naznin,[†] Salvatore La Cavera III,[†] Richard Cousins,[‡] Fernando Pérez-Cota,[†] Richard J. Smith,[†] and Matt Clark[†]

†*Optics and Photonics Group, University of Nottingham, University Park, Nottingham, NG7 2RD, United Kingdom.*

‡*Nanoscale and Microscale Research Centre, University of Nottingham, University Park, Nottingham, NG7 2RD, United Kingdom.*

E-mail: rafael.fuentesdominguez1@nottingham.ac.uk

Fabrication

Here, a grid pattern (2x2 mm) was fabricated on top of the glass substrate by a photolithography process. In this way, the region of interest could be imaged in the optical and SEM microscopes, so that the same imaging area and orientation could be easily found in all instruments. Additionally, the cover-slip was treated with an electrostatic self assembly layer-by-layer process to avoid the nano-structures being washed away when water is added.

The metallic nanostructures used in this manuscript are composed of rods and spheres. The gold nanorods (NanoPartz) are 145x50 and 112x40 nm in length and width, respectively, and the size variation is 10%. The gold spheres are 100 and 150 nm diameter (Sigma-Aldrich, 20% polydispersity), and 125 nm (NanoPartz, 4% polydispersity). All the previous values are provided by the manufacturer.

Mechanical vibrations of nanostructures

By measuring the vibrational frequencies of each metallic nanostructure using time-resolved measurements, we can extract the real size as well as the shape. These vibrational modes can be described with continuous mechanics which can be used for nanostructures made of more than a few hundreds of atoms. For a sphere, the following equation describes the main vibrational mode:

$$f_s = \xi \frac{c_L}{2R} \tag{S1}$$

where ξ is an eigenvalue that tends to 1 for the main vibrational mode and when there is a weak coupling between the particle and the medium, and c_L is the material longitudinal sound velocity and R, the radius.

In the case of a nanorod with cylindrical shape, two main vibrational frequencies are present, along the length and the width which are referred as extensional (eqn (2)) and

breathing mode (eqn (3)), respectively. These expressions are valid only if 2L/w≫ 1, where L and w are the length and width of the rod.

$$f_{ext} = \frac{1}{2L}\sqrt{\frac{E_{[100]}}{\rho}} \tag{S2}$$

$$f_{br} = \frac{\tau_0}{\pi w}\sqrt{\frac{E_{[110]}(1-\nu)}{\rho(1+\nu)(1-2\nu)}} \tag{S3}$$

where $E_{[100]}$ and E_{bulk} are the Young's modulus of the 100 crystallographic orientation and the bulk one, respectively; ρ, the density; ν, Poisson's ration; and τ_0 is the first eigenvalue given by:

$$\tau J_0(\tau) = \frac{1-2\nu}{1-nu} J_1(\tau) \tag{S4}$$

where J_0 and J_1 are the first two Bessel functions of the first kind.

By knowing the mechanical properties of nanostructures, we can extract their dimensions by applying the previous equations. For the calculation of the size and shape, the values in Table S1 were used.

Table S1: Values used to calculate the dimensions of gold nanostructures.

c_L	$E_{[100]}$	$E_{[110]}$	ρ	ν
3240 ms^{-1}	42 GPa	79 GPa	19700 kg m^{-3}	0.42

Although the surrounding medium can affect the vibration of the nanostructures due to damping, we do not measure any frequency shift experimentally whether the nanostructures are in air or water (see below figure, the vibration is damped strongly in water, however, there is not change in frequency). Therefore, we have assumed that the damping is not significant in our measurements to characterize the size and shape.

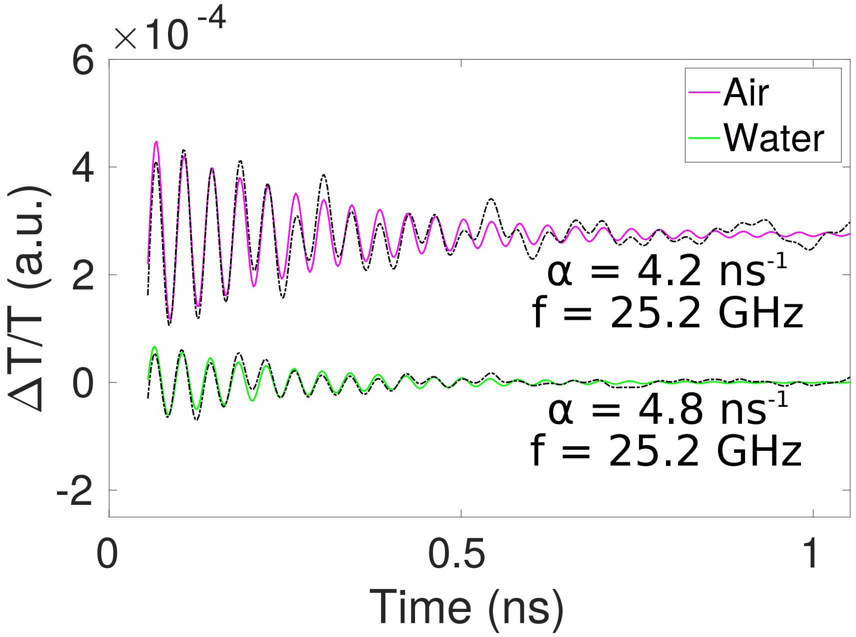

Figure S1: Time-resolved measurements of a 125 nm gold nanosphere in air and water. There is not significant frequency shift due to the surrounding environment.

Scanning electron microscope images

Prior to optical imaging the samples were imaged using a JEOL 7100F FEG-SEM electron microscope using a voltage of 15 keV, x19,000 magnification for the high resolution images and a working distance of 10 mm. The coverslips were overcoated with 80 nm of ITO prior to particle deposition in order to remove artefacts caused by charge build up. This step avoids the need of coating the whole sample with a metal layer (after deposition the particles) which will affect the acoustic data and the vibrational modes of the nanostructures.

Image reconstruction and localization

The image was reconstructed by identifying peaks in the Fourier spectra caused by the vibrating nanostructures. The frequency of vibration was used to identify the size and shape of the nanostructures and then the spatial distribution of the vibration amplitude at that frequency (a vibration point spread function) was centroided to determine the location.

A two stage centroiding algorithm was employed to reduce the contribution of noise from outised of the point spread function, first an initial centroid was computed using all the data, then a Gaussian mask (width approximately two optical point spread functions wide and not critical) was applied to suppress noise from outisde of the PSF and the centroid was recomputed.

Noise and localization error estimation

The noise contribution to the potential error in the centroiding was estimated by measuring the standard deviation of signal level in regions without nanostructures and using this as a measure of the signal noise. This error was then used with simple error propagation analysis to estimate the position error of the calculated centroids and these calculations were confirmed by simulation of signals with random noise added. This gave an estimation of the positional error for our system as 3 nm for the typical signals shown in the results. This was achieved by manually identifying a rough window location for each particle, centroiding to get a first estimate of the nanostructures and using this estimate to position a Gaussian mask (approximately twice optical point spread function size) to suppress noise from dark pixels outside the nanostructure position.

Polarization sensitivity to pump and probe

Figure S2 shows the extinction cross-section variation with respect to the polarization angle of pump (blue) and probe (red). The maximum optical response variation for the nanorods used in the manuscript is achieved by using the probe laser, in this particular case, 780 nm.

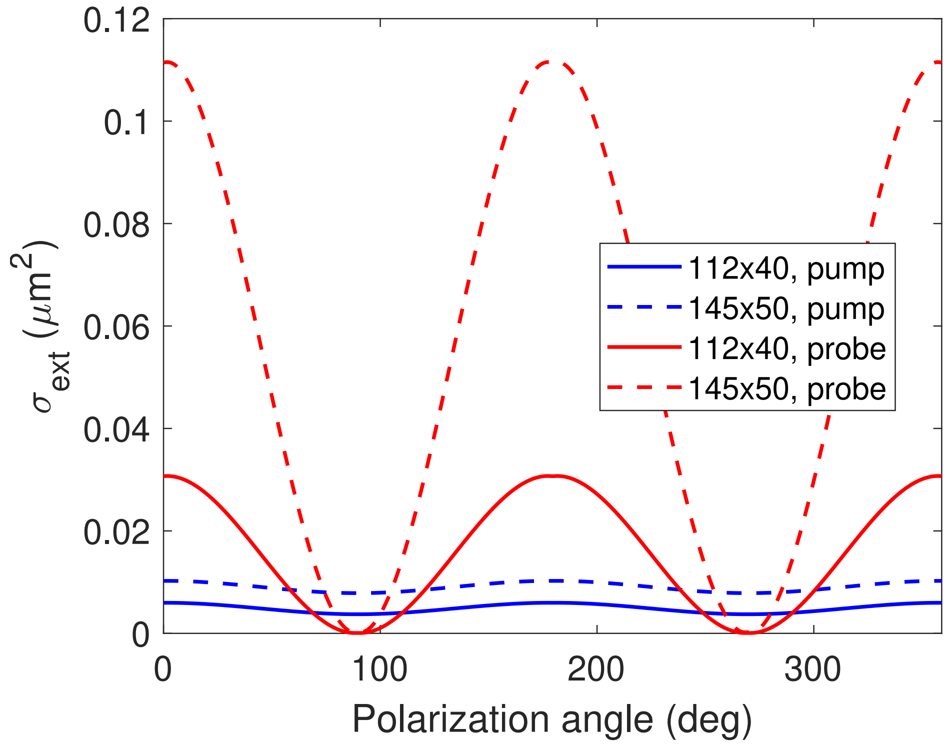

Figure S2: Extinction cross-section of 112x40 and 145x50 nm nanorods for linear polarized pump (blue) and probe (red) light when the orientation is 0 degrees. The maximum optical response variation is achieved by using the probe laser.

Experimental traces

Figure S3 shows the size, shape, position and orientation reconstruction of polarization sensitive time-resolved pump-probe spectroscopy. This also includes the time and frequency traces for each nanostructure and the angle orientation data.

Although the PSFs of two nanostructures shown in figure S3 might overlap in space, they are separated in the frequency domain. For example, a diagram showing the reconstruction of area (d) from figure 4 (in the main text) is below:

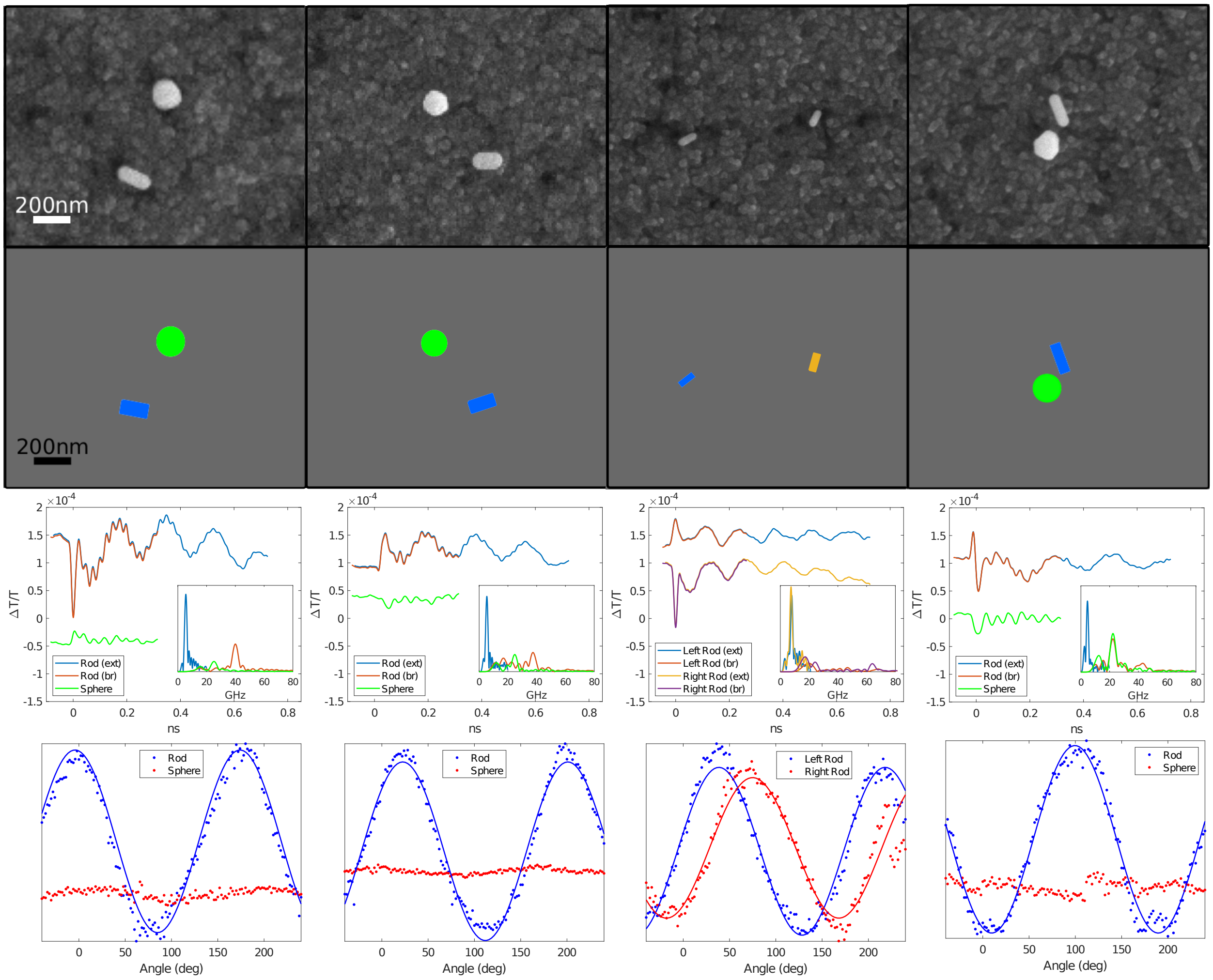

Figure S3: (First row) shows SEM images of four areas with multiple nanostructures inside the same optical point spread function of our experimental setup. (Second row) shows the super-resolution phononic reconstruction. (Third row) are time and frequency traces of each object. (Bottom row) are the FFT amplitude for different probe light polarization, where the maximum matches the nanostructure orientation.

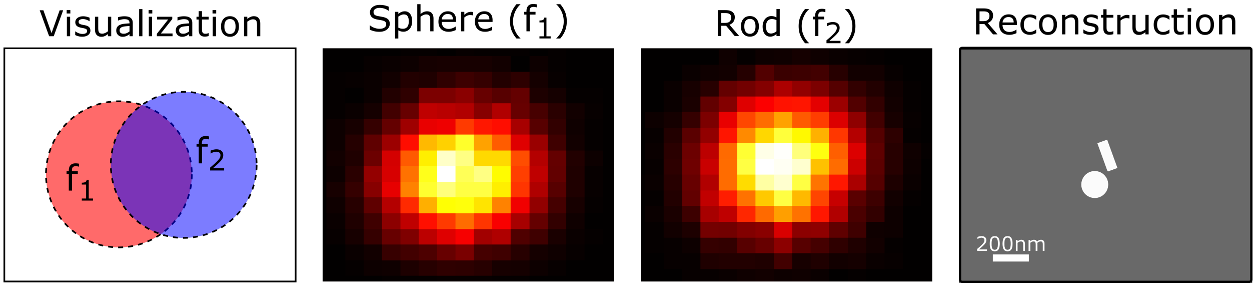

Figure S4: Visualization of two PSFs overlapping in space, but separated in the frequency domain. The sphere centroid is calculated from f1, and rod one from f2.
